# How Does Individual Psychotherapy Promote Recovery for Persons with Psychosis? A Systematic Review of Qualitative Studies to Understand the Patient’s Experience

**DOI:** 10.3390/bs14060460

**Published:** 2024-05-30

**Authors:** Laura A. Faith, Jaclyn D. Hillis-Mascia, Courtney N. Wiesepape

**Affiliations:** 1 Department of Psychiatry, Richard L. Roudebush VA Medical Center, Indianapolis, IN 46202, USA; 2 Psychosocial Rehabilitation and Recovery Center, Chillicothe VA Medical Center, Chillicothe, OH 45601, USA; jaclyn.hillis-mascia@va.gov; 3 Psychosocial Rehabilitation and Recovery Center, Austin VA Clinic, Austin, TX 78744, USA; courtney.wiesepape@va.gov

**Keywords:** psychosis, serious mental illness, psychotherapy, recovery, qualitative interviews

## Abstract

Psychotherapy for individuals with psychosis is an effective treatment that promotes recovery in various ways. While there is strong quantitative evidence across modalities, less is known from the patient’s perspective. There are many varied forms of psychotherapy, and gaining the patient’s perspective can improve understanding of salient elements of psychotherapy and increase engagement, ultimately improving recovery rates. The purpose of this review is to identify and integrate data from published studies of patient perspectives of psychotherapy for psychosis to understand essential elements across approaches, differences between approaches, and how psychotherapy impacts recovery. We aimed to understand further: what are the perceptions about individual psychotherapy from the perspective of individuals with psychosis? The current study was a systematic review using PRISMA guidelines of studies that included qualitative interviews with persons with experiences of psychosis who participated in psychotherapy. All three authors participated in the literature search using Pubmed, APA PsycInfo, and Psychiatry Online. We identified *N* = 33 studies. Studies included cognitive therapies, acceptance and mindfulness approaches, trauma therapies, metacognitive therapy, and music therapy. All studies reported participants’ perceived benefit with the therapeutic relationship as especially salient. Participants described diverse aspects of objective (e.g., symptoms, functioning) and subjective (e.g., self-experience or quality of life) recovery improvements, with perceived mechanisms of change, and with music therapy having some unique benefits. Participants also reported challenges and suggestions for improvement. Study findings highlight the salient aspects of psychotherapy identified by patients that may help therapists to individualize and improve approaches to psychotherapy when working with individuals experiencing psychosis. Overall, findings support the potential for integrative psychotherapy approaches for maximal treatment personalization.

## 1. Introduction

### 1.1. Psychosis and Treatment

Experiencing the onset of a serious mental illness (SMI) such as psychosis is a life-changing event that affects all areas of one’s life, including relationships, everyday functioning, sense of self, and one’s experience of the world [1]. Psychosis is a mental illness that affects people in various ways, and may include experiences of auditory or visual hallucinations, delusional beliefs, negative symptoms, cognitive challenges, and various functional difficulties [1,2]. We use the term ‘psychosis’ to include psychotic disorders with traditional diagnostic labels such as schizophrenia and schizoaffective disorder. This term will be used throughout our study as an intentionally broad term to encompass the experience of many individuals. Mental healthcare is important to help persons with psychosis return to their lives and find ways to engage in the world in a meaningful way. Individuals with SMI often utilize a range of mental health treatments that may address psychological distress, unremitting symptoms, loss of sense of self, or functional difficulty. For instance, psychosocial treatments, such as individual psychotherapy, may offer coping skills to manage symptoms, psychoeducation to understand diagnosis and treatment, and/or a dialogical space to navigate changes, gain perspective, and learn how to live with mental illness. National Institute of Clinical Excellence (NICE) guidelines recommend initiation of mental health intervention after the first episode of psychosis, including medication management, psychotherapy, and other psychosocial treatments [1,2]. While antipsychotic medication is considered a first-line treatment, some individuals may not want to take medications due to personal preference, lack of desired benefit, or potential side effects. Psychotherapy is a good alternative to medication, as it offers cognitive and behavioral mechanisms to allow individuals to manage their mental health through lifestyle changes, gaining understanding of oneself or others, and cognitive strategies. The vast majority of psychotherapy research for persons with psychosis included patients who were taking antipsychotic medication alongside psychotherapy; however, research has more recently explored the effectiveness of individual psychotherapy on its own [3,4]. Moreover, one recent study identified that immediate antipsychotic treatment after psychosis onset was associated with poorer five-year outcomes [5]. This suggests that there is a subgroup of patients who do not need immediate antipsychotic treatment and perhaps some that may benefit from psychosocial treatments such as psychotherapy as their primary treatment. It is important to continue to study individual psychotherapy as a fundamental and potentially stand-alone treatment for persons with psychosis, and to further understand the essential elements of psychotherapy that maximize its effectiveness.

### 1.2. Recovery after Psychosis

Recovery is a complex concept that has evolved alongside continued development of psychotherapy. Importantly, there is a distinction between objective recovery (i.e., outcome measured and defined by the clinician) versus subjective recovery (i.e., process measured and defined by the patient) [6]. Traditional models often focused on reduction or remission in symptoms and functional improvements within the scope of objective recovery. Grassroots movements in the 1980s and 1990s represented a paradigm shift that incorporated consumer definitions of recovery [7]. From this movement came the concept of subjective or personal recovery, which is a deeply personal process that involves changing one’s perspective, values, or roles. It is defined and assessed by the person experiencing psychosis themselves. It includes the development of life pursuits and meaning of one’s life [8]. Alongside the enhancement of recovery as a personal process rather than simply remission of symptoms, the aims of psychotherapy for individuals with psychosis have evolved to include more optimistic promotion of functional recovery and meaning-making [9].

As a more nuanced understanding of recovery developed, multiple psychotherapy modalities were developed with differing treatment foci and approaches. Cognitive Behavioral Therapy for Psychosis (CBTp) is one of the most well-established psychotherapies with numerous randomized-controlled trials that focuses on improving unhelpful thought patterns and promoting coping skills [10,11]. Acceptance and compassion-based treatments such as Acceptance and Commitment Therapy (ACT) and Compassion-Focused Therapy (CFT) are other forms of psychotherapy with a growing evidence base that focus on accepting psychological difficulties and improving compassion towards oneself [12,13]. Metacognitive therapies have emerged in recent years as other effective forms of psychotherapy. For example, Metacognitive Reflection and Insight Therapy (MERIT) is an integrative approach that helps individuals improve metacognition or understand themselves, others, and their place in the world so they can manage their mental illness, improve relationships, and improve quality of life [14,15]. Art and music therapy are forms of psychotherapy that utilize creative techniques to help people express themselves while understanding psychological and emotional aspects of art or music and how it might relate to themselves and their emotional experiences. These therapies are often offered in a group setting, but are sometimes offered as an individual psychotherapy [16]. Finally, as it is common for individuals with SMI to have experienced some form of trauma, there has been a resurgence in further understanding acceptability of trauma therapies for individuals with psychosis [17].

### 1.3. The Importance of Qualitative Research in Understanding Recovery

Traditionally, quantitative methods have been favored when researching the effectiveness and impact of psychotherapy for individuals with psychosis, most often using objective recovery definitions and measurement (e.g., symptoms). Quantitative research is helpful with gaining precise measurements to understand the degree of change in desired psychotherapy outcomes. Qualitative research is another potentially valuable avenue to gain important information about psychotherapy with data from interviews, for example, by understanding themes or patterns across interviews to understand the patient’s point of view. However, it is used less often, perhaps because has been regarded as “unscientific” or less rigorous than quantitative research [18]. This has led to a lack of research focused on the perspective of the patient and their experiences of psychotherapy. Without the patient perspective, clinicians may risk having a one-sided view of the important elements of psychotherapy, as studies have traditionally been designed and interpreted by researchers without asking the patient themselves what they think. Consistent with the paradigm shift from objective to more personal and pertinent aspects of an individual’s life, as well as to better understand the consumer’s definition of recovery, qualitative measurement can better capture the process of subjective recovery from the perspective of the patient themselves. In fact, researchers have noted that often times there is great discrepancy between what patients and therapists value in the working alliance, and therapists are only accurately able to identify what the patient believes are the most impactful moments in sessions about one-third of the time [19]. In addition, compared to therapists, patients appear to place greater emphasis on helpfulness, joint participation in the work of therapy, and negative signs of the alliance [20].

Recently, the importance of qualitative research has started to gain traction [21]. We propose that one way to further understand important factors relevant for a range of psychotherapy approaches is by examining the patient perspective across approaches. It is important to know how patients perceive psychotherapy and how it impacts diverse aspects of recovery. There is some evidence to support that patient perception of treatment could affect both engagement [22] and outcomes [23]. Qualitative studies about psychotherapy can complement quantitative studies by adding rich, contextual, and in-depth accounts of a person’s experience that can further enhance the breadth of information afforded by quantitative studies [18]. Interviewing patients allows them to speak in their own voice to best contribute to the development and improvement of treatments, and helps investigators compare their own perception of reality and definitions of recovery using the patients’ ideas. Furthermore, subjective and objective measures of functioning can offer different aspects of recovery from the clinician and patient that can be integrated to provide a more comprehensive picture [24]. Identifying the most salient aspects of treatment for patients may help better distinguish the different mechanisms of change contributing to recovery and further improve treatments.

### 1.4. Aims and Scope

The purpose of this review is to identify and carry out an integrated analysis of qualitative studies that include interviews of patients experiencing psychosis who participated in some form of individual psychotherapy. We aimed to understand further: what are the perceptions about individual psychotherapy from the perspective of individuals with psychosis? We hoped to include a wide range of studies with diverse modalities and approaches to understand broadly how individuals with psychosis experience individual psychotherapy. To our knowledge, this is the first published review with this focus. Holding et al. (2016) [25] published a review that synthesized individuals’ experience of psychological therapy for psychosis, although they included individual psychotherapy, the review was not focused on individual psychotherapy specifically and the majority of studies were group or family therapy. Additionally, Wood et al. (2015) [26] published a review of qualitative studies exploring patients’ experiences of CBT for psychosis. Distinct from these and other previous reviews, this is the first systematic review the authors are aware of that specifically aims to explore individual psychotherapy, across modalities, from the perspective of persons with psychosis. It is important to review diverse psychotherapy approaches to identify and further understand any similarities and differences across modalities. Specifically, we aim to integrate the findings of these articles to further understand, from the perspective of people with psychosis: (1) reported benefits of psychotherapy and how psychotherapy impacts recovery; (2) the experience of the psychotherapy process and what contributes to outcomes; and (3) critiques or areas of need. We intended to include a broad inclusion of studies as this is the first study of its kind and we aimed to gain a comprehensive understanding of patient experience from our findings. We hope these findings will improve our understanding of the essential elements of psychotherapy that promote recovery for persons with psychosis.

## 2. Method

### 2.1. Approach and Design

The current study was a systematic review using guidelines from the Preferred Reporting Items for Systematic Reviews and Meta-Analyses (PRISMA) [27,28]. Our research question was to understand the perceptions of individuals with psychosis who participated in individual psychotherapy. We intended to conduct a broad search to understand a range of psychotherapy approaches and how they impact persons with psychosis.

### 2.2. Study Eligibility and Exclusion Criteria

Qualitative studies consisting of interviews with adults aged 18 or older with psychosis (i.e., with a diagnosis of schizophrenia, schizoaffective disorder, or other schizophrenia/psychosis spectrum disorder) who participated in individual psychotherapy were included. We used the American Psychological Association’s (APA) definition of psychotherapy as a guide: “any psychological service provided by a trained professional that primarily uses forms of communication and interaction to assess, diagnose, and treat dysfunctional emotional reactions, ways of thinking, and behavior patterns” (American Psychological Association, 2024) [29]. We included studies related to all forms of individual psychotherapy (e.g., psychodynamic, cognitive, behavioral, humanistic, art/music, and integrative) with any duration of treatment. Eligible studies included at least one primary aim related to exploring patient perspectives of psychotherapy. This criterion was intentionally broad to capture a range of diverse study aims. We included studies with adolescent aged participants (e.g., early psychosis) if the study also included adults. Studies that included a range of diagnoses (e.g., a serious mental illness population) were included if they included individuals with psychosis or schizophrenia spectrum disorders within the sample. Studies with a comparison group or alongside clinician interviews were included as long as they included patient data. We were inclusive of additional participant demographic factors (e.g., we did not exclude based on lower or upper age limits, primary diagnosis, medication status, etc.). We included studies from all years of publication, with no restrictions on year of dissemination.

Studies that analyzed data from therapy which was not individual psychotherapy were not included, for example group therapy, family therapy, or case management. Studies investigating smart phone apps or other self-directed interventions (i.e., without a primary therapist as a major feature of the intervention) were not included. Studies that did not include individuals with a diagnosis of a primary psychotic disorder were not included, for example non-SMI diagnoses, clinical high risk for psychosis, or postpartum psychosis. Studies that aim to understand a very specific aspect of therapy (e.g., case formulation) or more broad aspects of recovery that were not specifically focused on psychotherapy (e.g., how psychiatric treatment affects recovery) were not included. Case studies were not included. Systematic reviews and meta-analyses were excluded. Unpublished manuscripts and conference abstracts were not included. Articles that were not in the English language were not included.

### 2.3. Search Strategy and Article Selection

Three groups of search terms were included: (1) “psychosis”, OR “schizophrenia”, OR “schizoaffective disorder”, OR “serious mental illness”, OR “severe mental illness”; (2) “qualitative”; (3) “psychotherapy” OR “therapy”. In terms of PICOS guidelines for searches [30]: Population: individuals with psychosis (i.e., with a diagnosis of schizophrenia, schizoaffective disorder, or other schizophrenia/psychosis spectrum disorder); Intervention: individual psychotherapy as defined by APA guidelines above; Comparison: qualitative data; Outcome: patient perceptions; and Study Type: qualitative method. Taken together, the research question is: Using qualitative data/method, what are the perceptions of individual psychotherapy from the perspective of individuals with psychosis? The quality of studies was assessed by examining how well each study met the inclusion criteria with an examination of the study’s aims, methodology, and patient sample by screening each title, abstract, and full text (if the title and abstract met inclusion criteria). Manual extraction was completed by all three authors for the first search using Pubmed. Search filters were applied for second (APA PsycInfo) and third (Psychiatry Online) searches by all three authors. Filters included: academic journals, qualitative study, interview, and English language.

**Figure 1:**
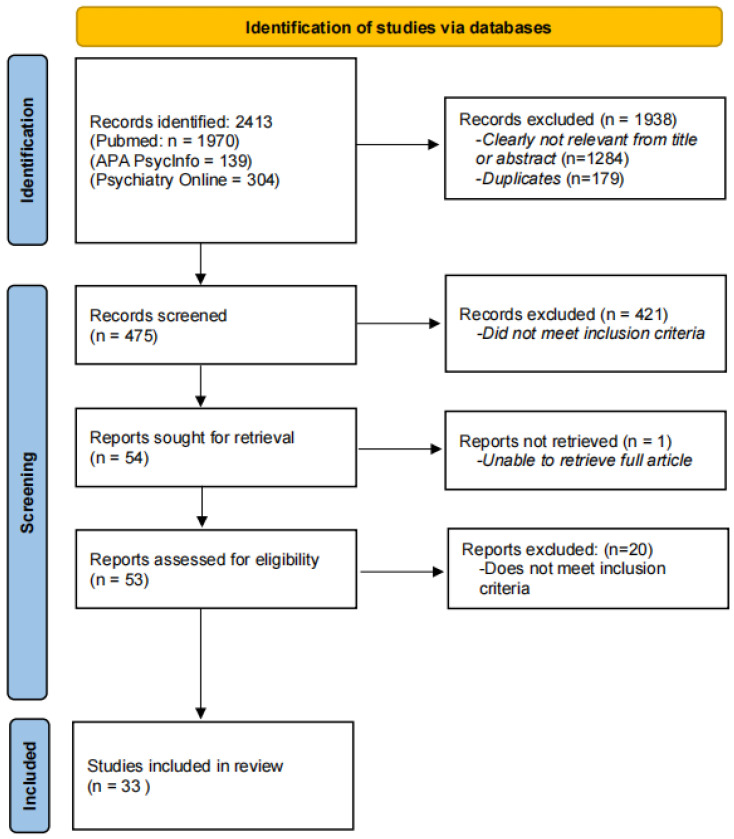
Flow chart of study extraction process.

The search was conducted between October 2023 and March 2024 by all three authors. The date of the last search was 11 March 2024. Studies identified from searches were examined to ensure they met inclusion criteria, and consensus was reached for the final list. There were *N* = 2413 records identified across all three databases. Records were excluded due to not being relevant or duplicates (*n* = 1938). There were *n* = 475 screened, of which *n* = 54 were sought for retrieval as potential articles to include in the review (*n* = 421 did not meet inclusion criteria). There was *n* = 1 that the authors were unable to retrieve and *n* = 20 that did not meet inclusion criteria. All discrepancies were discussed among all three authors throughout the process. Studies included in the review included *n* = 33 articles. See Figure 1 for a detailed depiction of the study extraction process.

### 2.4. Data Extraction and Quality Assessment

All three authors conducted independent extraction of study characteristics, methodologies, and results, as well as assessment of the limitations of each study. Any discrepancies were reviewed by at least two authors as they arose. No automation tools or software were used. The sequence for data extraction proceeded as follows: first author (year of publication), country, type of therapy (# of sessions), participants and setting, aims, analysis, results/themes (subthemes). Setting of the study varied between included studies and was reported as the most specific value that was available. Reporting of the number of sessions varied between included studies, as such data are reported as a range when included, and otherwise as a mean value (when range was not available). LF manually collected data to be integrated, and the second and third authors checked the consolidated data for accuracy and consistency.

After studies were identified, a summary of findings including characteristics of studies and findings across studies was completed by LF, reviewed by the second and third author, and is presented to summarize results. We will describe psychotherapy approaches, patient demographics, and methodologies used. Findings will be described to summarize similarities and differences across studies.

The authors did not use any automation tools to assess risk of bias. To reduce risk, all three authors conducted independent searches using three search tools. After the first author consolidated the data, the second and third authors checked the data for accuracy and consistency to reduce bias. This was a qualitative review, so effect size and other statistical bias are not relevant. The majority of our studies only included one group, so there was not randomization considered. We were interested to understand patient’s perspective of psychotherapy, which can be achieved with a single group design. However, comparison groups could add more information and potentially reduce bias. The authors considered limitations of the different studies as a measure of quality.

### 2.5. Data Synthesis

All data were integrated using the qualitative and thematic data from included studies. Results from each study were evaluated by reading qualitative data line-by-line and reporting patterns or common themes across data. A summary overview of results, including characteristics of the data as well as themes, is included in the Results Section. Common themes across studies were found by evaluating themes within each study, and reading content of each theme to gain cohesive understanding of themes and identify patterns of themes across studies.

## 3. Results

After a thorough search using the above-stated search criteria, 2412 articles were initially identified via the databases. Following the removal of duplicates and articles that did not meet inclusion criteria, a total of 33 articles were selected for this review. The PRISMA flow diagram (for more details, see Section 2.3. Search Strategy and Article Selection) illustrates the exclusion of studies at each selection stage (Figure 1). The 33 full-text articles were included in the review to follow; their suitability was verified according to predefined inclusion and exclusion criteria and then subjected to data extraction (Table 1 and Table 2) and quality assessment.

### 3.1. Study Characteristics

There were *N* = 33 studies that met inclusion criteria presented in Table 1 [31,32,33,34,35,36,37,38,39,40,41,42,43,44,45,46,47,48,49,50,51,52,53,54,55,56,57,58,59,60,61,62,63]. Publication years ranged from 2003–2023. The majority of studies were recent, *n* = 20 (60.61%) that were from the past 6 years, from 2019–2024. All studies included qualitative data with reported themes, including interpretive phenomenological analysis, thematic analysis, discourse analysis, or grounded theory. Inductive phenomenological analysis explores how individuals make sense of their experiences. Thematic analysis focuses on themes/patterns within the data. The one study reporting discourse analysis described their interest in how the client positioned themselves in relation to the therapist. Grounded theory is concerned with using qualitative data to generate theories. The current review focused on each study’s reported themes. Regarding modality/approach of psychotherapies included, the most common specific modality reported was CBTp (*n* = 3) [48,51,56] or other types of cognitive therapies (*n* = 5) [33,36,46,49,60]. Beyond cognitive therapies, other evidence-based approaches were included. There were three studies that analyzed Metacognitive Reflection and Insight Therapy (MERIT) [40,52,54]. There were three studies that analyzed Acceptance and Commitment Therapy (ACT) [32,37,39] and one with compassionate imagery [43]. Several additional studies involved a form of trauma therapy, including one study each of eye movement desensitization and reprocessing EMDR [41], trauma-focused imaginal exposure [42], prolonged exposure [47], narrative exposure therapy [55], and two studies including trauma-integrated psychotherapy for psychosis (TRIPP) [61,62]. In addition, two studies analyzed a technology-enhanced/assisted psychotherapy: Slowmo [45] and virtual reality therapy for negative symptoms [38]. There were three studies analyzing music therapy [31,57,59]. Two studies focused on hearing voices, including Talking with Voices [53], Making Sense of Voices [58], and Relating Therapy [50]. Other studies tested newer approaches including interventions the Feeling Safe Program [35], Social Recovery Therapy [44], and Low-Intensity Psychological Therapy [63]. One study included participants who engaged in differing psychotherapy modalities [34].

The majority of studies included a cohesive sample of persons with psychosis, schizophrenia, or schizoaffective disorder [32,33,34,35,36,38,40,43,44,45,48,49,50,51,52,53,54,56,60,61,62,63]. There were twelve studies which included a broader population of serious mental illness that included additional diagnoses (e.g., depression with psychotic features or post-traumatic stress disorder) along with psychosis [31,39,41,42,46,47,55,57,58,59,61,62]. One study included individuals with clinical high risk in addition to the individuals with early psychosis that fit inclusion criteria, thus it was included in the analysis [37]. The majority of studies were conducted in the United Kingdom [31,33,35,38,39,43,44,45,46,48,49,50,51,53,56,60,63], followed by Australia [32,42,61,62], the United States [36,47,52,54], The Netherlands [37,40,55], Norway [34,59], Germany [58], New Zealand [41], and South Africa [57]. The number of study participants ranged from four to twenty-five.

### 3.2. Aim 1: Perception of Benefit, Impact on Recovery, and Mechanisms of Change

Overall, participants across studies had positive views of psychotherapy and found it beneficial to their recovery in various ways. All studies included themes related to improvements or benefits following engagement in or completion of psychotherapy. One of the most salient benefits mentioned in all of the studies included in the review related to objective recovery outcomes, including improved symptoms, functioning, or achievement of goals (e.g., related to the specific psychotherapy presented, such as gaining control over voices) [32,33,34,37,38,39,40,41,42,43,45,47,49,50,51,52,54,55,56,58,59,61,63]. Studies also included themes related to improvements in subjective areas of recovery, for example, improved quality of life, sense of oneself or one’s identity, or self-confidence or self-compassion [31,34,35,40,41,42,44,45,52,53,54,55,57,59]. Others noticed a different relationship with the world overall [34,42,45] Participants also discussed specific parts of therapy that seemed to drive changes (i.e., mechanisms of change). This included improved agency or feeling in control [33,44,46,54], as well as new ways of thinking or meaning-making [35,39,46,48,54]. Others mentioned enhanced understanding of oneself [44,54,55], their illness, or their symptoms [39,53,56,60], or learning new skills (e.g., coping skills, tools, or intervention specific activities) [39,60,63]. Some mentioned emotional expression as cathartic [48], fulfilling [57], and a source of connecting with self and others [59].

### 3.3. Aim 2: The Experience of Psychotherapy

Regarding overall experience of psychotherapy, the most salient theme was reflection on the therapeutic relationship. Overall, participants had positive views of the relationship and found it to be instrumental in psychotherapy and recovery. Several mentioned that the therapeutic relationship felt similar to a friendship [31,34,35,55,56] and that it was an equal/collaborative relationship [31,56]. Important qualities participants mentioned included that their therapist was supportive and nonjudgmental [33,48,53,55,60]. Other descriptions included feeling acceptance and to have someone they were comfortable with and they could trust [35,45,48]. They felt it important to be respected and understood by their therapist [34,40,48,49,56]. They valued having someone who was flexible, personalized therapy, and listened to them [35,45,48]. Participants valued a therapist who cared about them and their life story [56]. Participants valued a therapist who challenged them [44,46], although some were off put when they felt the therapist was skeptical or questioning the validity of their thoughts [33]. One study with forty sessions mentioned that the timeframe helped them have adequate time to get to know and trust their therapist [40]. Another study reported that having a shared reality or language was helpful in therapy [34]. In a few studies, participants mentioned distrust in the therapist or lack of support as a barrier to psychotherapy [33,36,55]. For example, not feeling listened to sometimes led to lack of trust [49]. These were more often mentioned in studies with briefer interventions.

In general, participants experienced psychotherapy as challenging yet rewarding. Several studies reported participants as skeptical of psychotherapy at first, but felt it beneficial once they engaged [33,41,42,47,49]. Some studies reported that participants mentioned that psychotherapy was intense or emotionally burdensome [33,41,48]. At times, participants reported that psychotherapy brought distress to the surface as something to be worked on [44,48], yet at other times this distress was difficult for participants to tolerate [47]. Some reported that psychotherapy did not help or made symptoms worse [33,47,48].

Studies analyzing music therapy reported some participant experiences that were distinct from other modalities included in this study [31,57,59]. Participants valued creativity and found the intervention to be emotionally fulfilling. Participants felt a sense of freedom/liberation that was positive for them. At times, this meant freedom from symptoms, but often it was broader than that; music was a freeing experience that gave participants a sense of liberation within themselves during the process of music therapy. They were able to explore parts of themselves outside their illness that gave them a sense of identity and purpose. They were able to unleash creativity, playfulness, and express themselves. The self-expression was unique in that it was not limited by words as traditional talk therapy is, and it was a new way of self-expression for many. Participants described music therapy as therapeutic but different than traditional mental health treatment, and that it was distinct from other types of individual therapy. Participants also described music therapy as giving them greater connection to others and the world. For some, this was related to their connection with the therapist and engaging in an activity together as equals. Others mentioned that music therapy helped open an avenue for greater connection to others in the world (e.g., friends and family), attending concerts, or joining a local choir or band.

### 3.4. Aim 3: Critiques of Therapy

Several studies included participant critiques or suggestions for the psychotherapy. Participants often mentioned a need for personalization and flexibility when there was a perceived lack thereof [33,36,37]. Several studies reported participants desiring more time or feeling as though the therapy ended abruptly [36,38,39,48]. Some studies reported participants as having difficulty understanding concepts [37,39], technical issues [38,45], treatment as too structured, or not feeling as though the treatment was a good fit [37,39].

For a summary of common themes, see Table 2.

## 4. Discussion

The current study was a systematic review that included qualitative interviews with patients with psychosis who participated in individual psychotherapy. We aimed to ingrate data from qualitative studies to further understand the perceptions of individuals with psychosis who participated in individual psychotherapy. We included studies from a range of different approaches, including cognitive therapies, acceptance and mindfulness-based therapies, metacognitive therapy, trauma treatments, and music therapy. More specifically, we sought to understand: (1) reported benefits of psychotherapy and how psychotherapy impacts recovery; (2) the experience of the psychotherapy process and what contributes to outcomes; and (3) critiques or areas of need.

### 4.1. Psychotherapy Benefit on Objective and Subjective Recovery, and Mechanisms of Change

Participants in the included studies reported that psychotherapy helped with a range of distinct recovery outcomes, including both objective and subjective aspects of recovery. As expected, participants reported improvements in objective aspects of recovery, including symptoms, goals, and functioning. Approaches that emphasized objective recovery were those that were more structured and targeted symptoms or psychosocial functioning, such as CBT, ACT, and trauma therapies. Participants also reported improvements in subjective aspects of recovery such as sense of self, quality of life, self-compassion, and relationship with the world. Across all three Metacognitive Reflection and Insight Therapy (MERIT) studies, themes related to self-understanding were reported, including self-compassion, self-expression, and self-esteem [40,52,54]. Unique from many approaches included here, MERIT incorporates the development of understanding the narratives of one’s life to improve their sense of self and narrative identity [64]. Interestingly, one study reporting results from narrative exposure therapy, a type of trauma treatment for people with psychosis, also reported patient experience of increased self-knowledge through improved understanding of one’s life [55], supporting the idea that eliciting life narratives is an essential element in promoting self-understanding.

Participants noted aspects of psychotherapy that they thought contributed to change (i.e., mechanisms of change). The concept of agency appeared across different modalities as an important mechanism of change. Patients described the value in being able to take control of their lives and gain valuable skills/knowledge to gain mastery over difficulties. Agency is essential for individuals to manage mental health and decide how to engage in their lives beyond their illness. However, some individuals may have difficulty directing their own activities for example due to lack of motivation or negative symptoms. In this case, patients may need extra support to bolster agency. For instance, one study outlined how metacognitive intervention was essential for a patient to later engage with behavioral activation [65]. Other themes related to mechanism of change included gaining insight, a new way of thinking, and emotional expression. It is not surprising that these were the reported mechanisms of change, as cognitive and emotional work is an important focus of transtheoretical approaches, such as CBT, ACT, trauma, and metacognitive approaches. Lastly, learning skills was mentioned as an important part of therapy, for example, coping as a part of managing distress.

These findings provide further support for the idea that both objective and subjective aspects of recovery are important and should be assessed in psychotherapy [6]. It is possible that that different approaches may emphasize differing aspects of recovery, although it seems important to include a range of processes and outcomes to fully assess the effect of psychotherapy for the patient.

### 4.2. Therapeutic Relationship

Not surprisingly, the therapeutic relationship was reported as an essential part of psychotherapy across modalities. Participants described the alliance as helpful in working through difficult topics in therapy, and that once they trusted their therapist, the work was more meaningful/effective. This finding aligns with research suggesting that the therapeutic relationship or working alliance is an important predictor of treatment outcome [66]. Moreover, it has been suggested that often the patient’s experience of the working alliance is more predictive of successful outcomes of psychotherapy as compared to the clinician’s perspective [19]. One recent meta-analysis used to examine both patient and therapist perspective on the therapeutic alliance among individuals diagnosed with schizophrenia spectrum, personality, and substance use disorders found that patients across all three diagnostic groups tended to estimate the therapeutic alliance as somewhat higher than did their therapists on quantitative measures [67]. Conversely, a few studies reported negative alliance, which illustrates that when alliance is poor, it has a detrimental effect on the experience of treatment. This aligns with previous studies that aim to understand how poor alliance affects psychotherapy. For instance, one study with male clients found that negative alliance was associated with rigid adherence to a therapeutic approach that was not a good fit for the patient [68]. In other words, the approach was inconsistent with the patient’s view of what was most helpful, important, or relevant.

In addition to the establishment of a positive therapeutic relationship and basic trust, understanding the patient’s subjective experience of therapy might be an essential factor to help facilitate recovery. For example, clinicians may benefit from promoting reflection about the patient’s perspective on the therapeutic alliance and the processes that are occurring within the therapy itself in order to integrate the patient’s feedback into ongoing therapy. One study found in particular found that using interventions to promote reflection on the progress in therapy within a given session was found to be correlated with higher scores on a measure of therapeutic alliance [69]. Research further suggests that when the therapist is willing and open to seek the patient’s perspectives and are non-defensive in accepting negative feedback, it is likely to contribute to a stronger alliance [70]. Thinking together and encouraging the patient to openly reflect on their ideas about the therapeutic alliance, their experience of what it is like to be in the presence of the therapist, and also what might they believe contributes to their own growth process, may help the therapist to gain a greater appreciation for the difficulties the patient is facing and working to overcome. This would allow for the therapist to gain perspective of the patient’s subjective experiences, fill any potential gaps between the patient and therapist’s perspectives, and ultimately, encourage the patient to better understand themselves.

### 4.3. Challenges/Barriers and Critiques of Psychotherapy

There were several challenges and criticisms of psychotherapy for people with psychosis that are important to consider. Several studies mentioned that participants found psychotherapy to be additionally distressing and they did not have the opportunity to resolve this distress during the course of treatment. It was also reported that some found psychotherapy to be too difficult in either content or structure. In addition, several studies reported that participants mentioned treatment being too brief. It is standard for evidence-based psychotherapy to be offered in the form of 12 sessions or less, and several studies included in this review followed this structure. However, there is evidence that psychotherapy for persons with psychosis should offer at least 16 sessions for significant improvement, and there is often greater benefit with increased number of sessions [71,72]. It is likely that patients vary in the number of sessions that is required as the “correct dose”, as such treatment providers should allow flexibility, when able, in an individual’s treatment plan. It is possible that an incorrect dose (i.e., not enough sessions) of therapy may contribute to unresolved distress that is increased as therapy begins and there is inadequate time to allow for distress to resolve. Important to consider are integrative treatments that could be useful to offer a range of content and structure to best individualize treatment [73]. For example, if a person needs a greater number of sessions or additional processing time to manage distress. Further, an integrative approach can bring together theories and approaches to support recovery, which is especially important, as personal recovery can have different meanings for different people [74]. As such, there are several models of integrative treatment that propose utilizing elements from existing theoretical models such as cognitive, interpersonal/intersubjective, developmental, psychodynamic, and metacognitive approaches [75,76,77,78,79].

### 4.4. Unexpected Findings: Unique Impact of Music Therapy and Similarities to Other Approaches

We included studies exploring patients’ views of music therapy, and findings suggest that this modality has a distinct impact on patients compared to other psychotherapy approaches. While traditional psychotherapy approaches focus on assisting individuals with coping strategies or understanding an identified psychological problem through talk therapy (e.g., positive symptoms, trauma memories, sense of self), music therapy is a distinct therapeutic method that uses music as an intervention to accomplish goals (e.g., emotion regulation, social skills, self-esteem) [80]. Participants who engaged in music therapy expressed value in the ability to be creative and feel free from psychiatric symptoms while engaging with music. Thus, music therapy may allow patients to explore aspects of themselves outside of psychiatric symptoms so they can improve their sense of self, gain confidence, and remember who they are despite their diagnosis. It might be that music therapy allows patients to better identify and express aspects of themselves that are difficult to communicate otherwise (e.g., in the form of words and verbal language) and thus non-traditional therapy approaches may provide unique benefits for patients and should be considered as part of a treatment plan. Importantly, music therapy may be able to inform talk therapy approaches to widen their treatment targets and improve the intersubjective space by integrating creativity within the therapy approach. For example, the use of play has been discussed as a means to allow the patient to engage in “creative mutual exploration” [81] and a means to communicate with the self and others [82]. Clinicians can inform their practice with forms of art, for example, considering jazz as a psychotherapy framework which utilizes improvisation with timing, risk-taking, and having a flexible role/ego [83]. Music therapy thus may be unique in its opportunity to explore aspects of subjective recovery that are often less of a focus in other forms of talk therapy. It may address an important gap in more traditional approaches of psychotherapy, which can sometimes risk reducing recovery to symptom reduction or alleviating dysfunction without allowing for a strengths-based model [84].

### 4.5. Limitations

The current study has limitations worth noting. We included a range of different studies with varied therapeutic approaches and varied qualitative methods. While this adds diversity to our review, we recognize that it also contributes to a lesser degree of cohesion. The majority of studies included had modest sample sizes, allowing for depth in findings, but may contribute to limited generalizability. Additionally, most of the studies were from cognitive or trauma-focused therapies. More research is needed to contribute to understanding of patient’s experience of other modalities such as psychodynamic, humanistic, or integrative approaches.

### 4.6. Summary and Conclusions

In summary, participants reported psychotherapy, in its many forms, as beneficial to diverse aspects of recovery, including both objective and subjective outcomes. They valued positive changes within themselves (e.g., changed thinking, skills obtained, improved insight, improved self-confidence), the therapeutic relationship, personalization, and flexibility in the treatment itself. Some of the important elements included were therapeutic alliance, learning, working through fears/challenges, sometimes referred to as common factors, which are consistently considered to be important aspects of therapy [85,86]. Importantly, experiences of psychotherapy for persons with psychosis varied greatly, supporting the idea that recovery is a complex process that can involve both objective outcomes and subjective, personal processes such as understanding oneself and one’s place in the world. It thus seems important when measuring progress and understanding recovery for persons with psychosis to have an integrative approach, by considering both objective, observable outcomes (e.g., reduction in symptoms, psychosocial functioning) and subjective processes (e.g., understanding of oneself and one’s life) [24,87].

This review has important clinical implications. We offer integrated findings from a range of psychotherapeutic approaches, further illustrating the diverse offerings for persons with psychosis. As each approach may be suited for certain individuals and psychological problems, and each person may have a different experience of treatment, it is important to continuously assess progress, subjective experience, and individualize treatment to best fit each person’s needs. As such, it is especially relevant to engage in shared decision-making practices with patients and determine a best fit for psychotherapy. For instance, determine with the patient what they are seeking in psychotherapy, whether or not they value structured learning, what they hope to achieve in psychotherapy, etc. Even when a single approach is used, there are factors that affect treatment outcomes such as third variables (e.g., insight) and common factors (e.g., the therapeutic relationship) [88] which may be gleaned from frequent feedback from the patient. For instance, it may be appropriate to designate session time for formal or informal feedback from the patient for qualitative or quantitative data including therapeutic alliance measures, recovery interviews, or how the patient understands changes resulting from psychotherapy. It seems important to highlight that integrative therapies may be a particularly helpful way to address the varied goals, needs, and accommodations for individuals seeking treatment. One example of this is Metacognitive Reflection and Insight Therapy (MERIT) [14], an integrative approach included in this review, which utilizes flexible elements rather than pre-determined sessions or agendas. One future direction includes further understanding and developing integrative therapy frameworks [9]. There are several effective offerings of psychotherapy for psychosis; it is likely that the most impactful approach for each person is one that is flexible and continuously adapts to integrate the unique needs of the person engaging in treatment.

## Figures and Tables

**Table 1:** Studies included in review.

First Author (Year of Publication)	Country	Type of Therapy (# of Sessions)	Participants & Setting	Aims	Analysis	Results/Themes (Subthemes)
Ansdell & Meehan (2010) [31]	UK	Music therapy (at least 10 sessions)	* N * = 19 adults aged 24–69 with chronic MH problems	To explore experiences of music therapy and its benefits and effectiveness.	Interpretive phenomenological analysis	Benefit goes beyond symptoms; reconnecting with music; working with music-health-illness narrative; therapeutic aspects of music are unified; musical processes are distinct; therapist as equal “musical companion”; music therapy is distinct from other therapies; benefits are compensatory or alleviatory; reinvigorated hope.
Bacon et al. (2014) [32]	AUS	Acceptance and Commitment Therapy (ACT) (8 sessions)	* N * = 9 people aged 16–65 diagnosed with psychosis in an OP setting	To investigate perspectives of therapeutic processes.	Thematic analysis	Usefulness of therapy; changes attributed to ACT; understanding of therapy; non-specific therapy factors.
Birchwood et al. (2018) [33]	UK	Cognitive therapy for harmful compliance with command hallucinations (CTCH) (25 sessions)	*N* = 25 adults aged 18–67 with psychosis from inpatient and OP settings	To explore service user experiences in the context of a randomized controlled trial to measure acceptability and tolerability.	Grounded theory, thematic analysis	Helpful aspects included gaining control over the voices, challenging the power/omniscience of the voices, the structured approach, normalization of hearing voices, and peer support. Concerns included anxiety about completing tasks, fear of talking to voices, need for follow-up/ongoing support and concerns with adaptability.
Bjornestad et al. (2018) [34]	Norway	Various approaches including CBT and psychodynamic frameworks (Average # of sessions was 76.3, range 2–416)	*N* = 20 fully recovered people aged 17–58 with diagnosis of psychosis or affective disorder with mood incongruent delusions	To explore the working ingredients of psychotherapy in the recovery process after psychosis.	Thematic analysis	Help with the basics; having a companion through chaotic turf; creating a common language; putting psychosis in brackets and cultivating wellness; building a bridge from psychosis to the world.
Bond et al. (2022) [35]	UK	Feeling Safe Programme (approximately 20 sessions)	*N* = 6 adults aged 22–62 with non-affective psychosis	To understand experiences of the intervention to inform future development using peer research methods.	Interpretive Phenological Analysis	Engaging with everyday life; openness, engagement, and personal responsibility; the therapist as a professional friend; gaining new knowledge and alternative perspectives; having the right pace in therapy; flexibility and fitting.
Bornheimer et al. (2023) [36]	US	Cognitive-behavioral suicide prevention (10 sessions)	*N* = 5 adults with mean age 44.7 with psychosis in community MH	To explore experience of receiving treatment (part of an open pilot study).	Grounded theory	Treatment experience; engagement enhancers; barriers to this treatment within community MH; and suggestions to improve CBSPp delivery in CMH.
Bouws et al. (2023) [37]	The Netherlands and Belgium	Acceptance and Commitment Therapy (ACT) (8 sessions)	*N* = 19 individuals aged 21–40 with clinical high risk or early psychosis from secondary MH services	To explore treatment perspectives for early psychosis.	Thematic analysis	Participants understood and connected with ACT, noticing more awareness and acceptance of their thoughts and feelings, and living more in line with their personal values. Suggestions included comments on personalization or not being psychosis specific enough and difficultly understanding material with active psychosis.
Cella et al. (2022) [38]	UK	Virtual Reality Therapy for the Negative Symptoms of Schizophrenia (V-NeST) (12 sessions)	*N* = 9 adults with mean age 37.1 with psychosis from community MH	To understand acceptability (part of a feasibility study).	Thematic analysis	Therapy goals; impact of the pandemic; issues with symptoms; using virtual reality; relevance of virtual reality; therapy procedures; suggestions for improvement.
Davies et al. (2019) [39]	UK	Acceptance and Commitment Therapy for Psychosis (ACTp)	*N* = 10 male service users aged 21–60 from a medium secure MH service with mixed MH diagnoses	To gain an understanding of experiences and changes.	Thematic analysis	Recovery (hope, helpful, improved relationships/support), insight (developing insight, normalization, reflective), developing skills (coping strategies, mindfulness, acceptance, and defusion, values and committed action), and accessibility (metaphors/visual prompts, format)
de Jong et al. (2020) [40]	The Netherlands	Metacognitive Reflection and Insight Therapy (MERIT) (40 sessions)	*N* = 14 adults with psychosis with mean age 46 in post-acute phase of treatment	To investigate experiences, effectiveness and related processes, similarities/differences from other interventions, and non-desirable factors/outcomes.	Grounded Theory	Clear changes including self, coping, interpersonal, cognitive, and affective. Factors contributing to change included: positive alliance, concrete problems, venting/self-expression, adherence/active role, and putting therapy into practice.
Every-Palmer et al. (2023) [41]	New Zealand	Eye movement desensitization and reprocessing (EMDR) therapy (maximum 8 sessions)	*N* = 10 adults aged 20–59 with PTSD and psychotic disorders who had received EMDR as part of a clinical trial, either in prison or in a hospital	To understand patient experiences of EMDR therapy while receiving forensic care.	Thematic analysis	Severe trauma was ubiquitous and greatly affected participants; participants initially reacted to EMDR with early skepticism; the therapy was initially emotionally taxing, but participants generally felt safe and persevered; they were pleasantly surprised by results (e.g., symptom reduction and personal transformation); EMDR fit with the forensic setting (empowerment in a disempowering place).
Feary et al. (2022) [42]	AUS	Trauma-focused imaginal exposure for hearing voices (6 sessions)	*N* = 10 people (age not reported) experiencing voices with mixed SMI diagnoses	To explore the experience of participants.	Thematic analysis	Benefits from the intervention (e.g., sense of self, decreased distress, wellness, understanding of symptoms/trauma); therapy was intense with later benefits; changes in relationship with the world; this therapy was a different approach with positives and negatives.
Forkert et al. (2022) [43]	UK	Compassionate imagery intervention (4 sessions)	*N* = 12 adults aged 18–65 non-affective psychosis and persecutory delusions from secondary MH services	To explore experiences of the treatment (as part of a feasibility study).	Thematic analysis	Effortful learning; seeing change (increased calm, clarity, and acceptance); taking it forward.
Gee et al. (2023) [44]	UK	Social Recovery Therapy (average 19.29 sessions) compared with treatment as usual	*N* = 19 people aged 16–35 participating in psychosis early intervention services	To explore how SRT was implemented and therapeutic processes.	Thematic analysis	Increased self-knowledge; relationship with therapist; facing your fears; pushing oneself, sense of achievement; increased agency; positivity, hope, optimism.
Greenwood et al. (2022) [45]	UK	SlowMo blended (therapist delivered and technology enhanced) digital therapy (8 sessions)	* N * = 22 adults aged 29–79 with psychosis and persistent distressing paranoia	To explore the experience of the therapy content and design.	Thematic analysis	Starting the SlowMo journey; the role of the therapist; slowing things down; value and learning from social connections; approaches and challenges of technology; improvements in paranoia and well-being.
Griffiths et al. (2019) [46]	UK	Transdiagnostic Cognitive Therapy: Method of Levels (MOL) (1–10 sessions)	* N * = 12 adults aged 19–62 with first-episode psychosis and mixed diagnostic presentation	To understand how people experienced the intervention (part of a feasibility randomized controlled trial).	Thematic analysis	The therapist’s approach; being in control; thinking and talking; gaining a different perspective about problems.
Grubaugh et al. (2017) [47]	US	Prolonged Exposure (PE) (10–15 sessions)	*N* = 14 adults with mean age 46.8 with a psychosis and PTSD	To understand patient reactions and responses to PE (part of a feasibility study).	Grounded theory	History of PTSD symptoms without previous treatment; initial hesitation of therapy and ability to manage difficult thoughts/emotions; treatment credibility; treatment benefits; intervention difficulties.
Hardy et al. (2022) [48]	UK	Trauma-focused CBT for psychosis	*N* = 6 adults aged 20–59 under the care of psychosis community MH teams	To understand experiences of therapy as part of a mixed methods study.	Inductive thematic analysis	Perseverance; establishing safety; the challenges of therapy; rebuilding one’s life after trauma.
Harris et al. (2023) [49]	UK	Cognitive Behavioral Suicide Prevention for psychosis (CBSPp) (maximum 24 sessions)	*N* = 20 adults with mean age 38 with non-affective psychosis from community MH	To investigate the views on the therapy (part of a randomized controlled trial).	Inductive thematic analysis	Affective attitude; burden; alliance; intervention coherence; perceived effectiveness; self-efficacy.
Hayward et al. (2018) [50]	UK	Relating Therapy (maximum 16 sessions)	*N* = 9 adults aged 24–61 years with psychosis	To explore the experience of participants.	Inductive Thematic analysis	Changes in me (feeling stronger, standing up for myself, connecting with others); changes in voices; and role plays.
Kilbride et al. (2013) [51]	UK	CBTp (# sessions not specified)	*N* = 9 adults aged 21–65 from either early psychosis intervention services or community MH team	To inform user-oriented perspectives on psychosis treatment.	Interpretive Phenomenological Analysis	Person-centered engagement; active process of structured learning; improvement of personal understanding; therapy is hard work; recovery and outcomes.
Kukla et al. (2022) [52]	US	Metacognitive Reflection and Insight Therapy (MERIT) (approximately 48 sessions)	*N* = 13 adults with mean age 44 with psychosis in an OP setting	To understand experiences of recovery outcomes.	Inductive thematic analysis	Improvements in real world functioning; increased formation of life pursuits; enhanced social connections; self compassion; improved quality of life and wellness.
Longden et al. (2023) [53]	UK	Talking with Voices (TwV) (12–39 sessions)	*N* = 13 individuals with psychosis	To investigate patient experiences of the treatment (part of a randomized controlled trial).	Inductive thematic analysis	Desire for appropriate help (motivation to reduce voice-related distress, limitation of other treatment options); engaging with voices (challenges, support/safety, exploration/revelation); contemplating the future (aftermath of adversity, living well with voices, resources).
Lysaker et al. (2015) [54]	US	Metacognitive Reflection and Insight Therapy (MERIT) compared with supportive therapy (approximately 48 sessions)	*N* = 25 adults with mean age 44 with psychosis from an OP VA Medical Center	To explore whether treatment can affect self-experience.	Thematic analysis	Both groups: improved confidence, self-esteem, clarity of thought, and goal setting. MERIT group: improved narrative coherence and personal agency.
Mauritz et al. (2022) [55]	The Netherlands	Narrative Exposure Therapy (NET) (5–16 sessions)	*N* = 23 adults aged 21–65 with mixed SMI diagnoses and comorbid PTSD from an OP setting	To understand patients’ experiences of NET concerning changes in symptoms, care needs, quality of life, and functioning.	Grounded theory	Increased awareness/worsening of symptoms during therapy with eventual improvement; improved self-knowledge, quality of life, and functioning; professional and informal support as helpful and needed during and after therapy (some reported this as adequate and others needed more); a few reported no improvements after treatment.
Messari, S. & Hallam (2003) [56]	UK	CBT for psychosis	*N* = 5 adults aged 28–48 with psychosis from inpatient and OP programs	To involve users in the planning and delivery of MH services.	Discourse analysis	This is truly happening; I am ill; CBT as an educational process; CBT as a respectful relationship between equals; CBT as a healing process; CBT participation as compliance with the powerful medical establishment.
Paul et al. (2020) [57]	South Africa	Music therapy (8 sessions)	*N* = 15 adults aged 18–57 with major depressive disorder (MDD) or an acute phase of psychosis from an inpatient psychiatric hospital	To explore patient reflections of music therapy.	Thematic analysis	Praise for music therapy; distress before/during therapy; opening up and emotionally dealing with old wounds; new perspectives; growing strong; emotional fulfillment; social closeness and more adept; liberation and creativity.
Schnakenberg et al. (2018) [58]	Germany	Experience-Focused Counselling (Making Sense of Voices) compared with treatment as usual	*N* = 9 adults aged 18–65 with psychosis or personality disorder	To explore whether Experience Focused Counselling could be considered trauma-sensitive.	Thematic Analysis	Trauma related; dealing with emotions; process of working with voices; intra- and interpersonal life; and coping related.
Solli & Rolvsjord (2015) [59]	Norway	Music therapy (14–55 sessions)	*N* = 9 adults aged 21–41 with psychosis or PTSD from an inpatient setting	To explore how patients experienced music therapy.	Interpretative phenomenological analysis	Freedom; contact; wellbeing; symptom reduction.
Taylor et al. (2019) [60]	UK	Cognitive Analytic Therapy (CAT) (4–28 sessions)	*N* = 4 adults aged 19–34 with psychosis from secondary care MH services	To understand the experience of receiving CAT (part of a case series study).	Thematic analysis	Insight into experiences; building a therapeutic relationship; the usefulness of CAT tools; making positive changes.
Tong et al. (2017) [61]	AUS	Trauma-integrated psychotherapy for psychosis (TRIPP) (unspecified # of sessions)	*N* = 8 adults aged 18–27 with comorbid first-episode psychosis and PTSD from an early psychosis intensive OP program	To explore young people’s reactions to a trauma-focused treatment for PTSD in FEP.	Interpretive phenological approach	Distress in session; relief in and out of session; symptom exacerbation out of session.
Tong et al. (2019) [62]	AUS	Trauma-integrated psychotherapy for psychosis (TRIPP) (unspecified # of sessions)	*N* = 11 adults aged 18–27 with comorbid first-episode psychosis and PTSD	To gain an understanding experiences of treatment and how experiences related to trauma-informed/specific treatment.	Interpretive phenological approach	Reluctance to approach the trauma memory (not wanting to talk about trauma, difficulty acknowledging the trauma, not wanting to re-experience trauma-related emotions); factors aiding the process (desire for change, not being pressured to talk, therapeutic relationship, time).
Waller et al. (2015) [63]	UK	“Low intensity” psychological therapy (8 weekly sessions with 1 booster at 1 month)	*N* = 17 adults average age 41.17 with psychosis who were in adult MH services	To evaluate the acceptability of the training protocol and the therapy, and to examine the factors promoting and restraining implementation.	Thematic analysis	Benefits of the intervention: learning new skills, achieving their goals, and therapeutic relationship. Barriers: interference of physical/MH problems and not crediting achievements. Suggestions for improvement: difficulty understanding material, not a good fit, and need for more sessions.

Note: MH = mental health; OP = outpatient; UK = United Kingdom; AUS = Australia; US = United States.

**Table 2:** Themes and subthemes across studies.

Theme	Subtheme
Improvements in objective recovery	Symptom reduction
	Functional improvements
	Achievement of goals
Improvements in subjective recovery	Sense of oneself
	Self-confidence/self-compassion
	Quality of life/wellness
	Engagement with the world
Mechanisms of change	Insight (understanding oneself/illness/symptoms)
	New/improved way of thinking
	Autonomy/agency
	Learning skills
	Emotional expression
Therapeutic relationship as an important part of psychotherapy	Equal/collaborative relationship
	Resembled a friendship/companionship
	Supportive and nonjudgmental
	Trust
	Respect and Understanding
	Flexible & Personal
	Challenging
Challenges/Barriers of psychotherapy	Problems with therapeutic alliance (e.g., lack of trust)
	Early skepticism of therapy
	Intense/emotionally burdensome
	Increased distress/symptoms
Critiques of psychotherapy	Need for personalization/flexibility
	Desire for more time
	Difficulty understanding topics
	Technical issues
	Treatment not a good fit (e.g., too structured)

## Data Availability

No new data were created or analyzed in this study. Data sharing is not applicable to this article.
